# Expertise in Evaluating Choreographic Creativity: An Online Variation of the Consensual Assessment Technique

**DOI:** 10.3389/fpsyg.2018.01448

**Published:** 2018-08-24

**Authors:** Lucie Clements, Emma Redding, Naomi Lefebvre Sell, Jon May

**Affiliations:** ^1^Department of Psychology and Counselling, University of Chichester, Chichester, United Kingdom; ^2^Dance Science, Trinity Laban Conservatoire of Music and Dance, London, United Kingdom; ^3^School of Psychology, Cognition Institute, University of Plymouth, Plymouth, United Kingdom

**Keywords:** creativity, choreography, contemporary dance, expertise, audience, assessment

## Abstract

In contemporary dance, experts evaluate creativity in competitions, auditions, and performances, typically through ratings of choreography or improvisation. Audiences also implicitly evaluate choreographic creativity, so dancers’ livelihoods also hinge upon the opinions of non-expert observers. However, some argue that the abstract and often pedestrian nature of contemporary dance confuses non-expert audiences. Therefore, agreement regarding creativity and appreciation amongst experts and non-experts may be low. Finding appropriate methodologies for reliable and real-world creativity evaluation remains the subject of considerable debate within the psychology creativity research field. Although considerably variant in methodological operationalisation, the Consensual Assessment Technique (CAT) asks individuals to use an implicit definition to assess creativity in others’ work. This study aimed to investigate the role of experience and expertise in the evaluation of choreographic creativity, with a secondary aim of testing the feasibility of an online snowballing methodology for large-scale dance-specific research, informed by the methodology of the CAT. We filmed 23 Contemporary Dance students each performing a 3-min peer-choreographed solo and then recruited 850 online evaluators with varying degrees of expertise and experience in dance and creativity. Evaluators viewed at least one randomly selected video and rated creativity, technical ability, appreciation and understanding of the work, each using a seven-point Likert scale. A one-way ANOVA showed a significant difference in creativity ratings across the 23 videos, and creativity correlated significantly with the other variables. We then categorized evaluators on nine aspects of their dance and creative experience and entered the data into a repeated-measures linear mixed model. Two of the fixed effects yielded differences in creativity evaluations: (i) contemporary choreographic experience and (ii) self-reported creative expertise, as did the random effect of the video. The results indicate that personal experience of the choreographic process impacts creativity assessment, above and beyond experience in dance class participation. Implications for creativity assessment within creativity research and practice are discussed.

## Introduction

‘Contemporary dance’ loosely refers to a range of dance styles that use the body to explore and express conceptual ideas or images (Strauss and Nadel, 2012). In contemporary dance, there are no set movement sequences to draw from so there is an expectation of finding new and inventive movement. The focus is, therefore, less on the formulaic construction of movement than in classical forms such as ballet, with an often-deliberate rebellion against codified technique. It is this freedom that supports the argument that contemporary dance is creative by nature (H’Doubler, 1998). Researchers commonly cite Guilford (1950) presidential address to the American Psychological Association as the defining moment in persuading psychology researchers of the value and importance of scientific research into creativity (Kaufman and Sternberg, 2010; Runco, 2014). Psychological research has facilitated depth of understanding of the predictors, correlates and consequences of creativity, but typically focuses on general population research, with less research within specialist domains (Kaufman and Sternberg, 2010; Runco and Acar, 2012; Long et al., 2014; Runco, 2014; Simonton, 2015). Little has been published drawing on scientific methods within the domain of contemporary dance (Thomson and Jaque, 2017).

The lack of creativity research within the performing arts more broadly may be due to scientists’ misinformed beliefs that performing artists are replicators who express work generated by others, rather than creators, and are therefore not a population of interest (Kogan, 2002; Sawyer, 2014; Thomson and Jaque, 2017). Butterworth (2004) notes that this traditional hierarchy of ‘choreographer-as-creative’ and ‘dancer-as-reproducer’ is no longer the sole means by which creativity occurs, citing numerous ways in which the dancer expresses their creativity in choreography. The boundary between dancer and choreographer is blurred, and dance students now learn both performance and creative skills. Professional contemporary dancers often contribute to the development of movement material, through ‘exploring, selecting, and developing dance material’ (Stevens and McKechnie, 2005, p. 40). The process is often guided by ‘tasking’, the use of a problem set by the choreographer, and solved by the dancers (May et al., 2011). Typically, each dancer’s material will contribute in some way, through refinement of the movement and changes to timing, resulting in a creative product (Stevens et al., 2001). Farrer (2014) notes that whether improvising, choreographing, transforming a phrase of movement, or completing a task, dancers embody numerous creative roles, yet even dancers themselves do not recognize their creativity. These multiple perspectives highlight a broad lack of awareness of dancers’ choreographic creativity, calling for greater scientific attention to this unique domain of creativity.

The purpose of our work was to investigate how experience in contemporary dance impacts assessment of choreographic creativity, because contemporary dance requires communication of creative ideas to an audience (Humphrey, 1959; Burrows, 2010; Risner, 2000). Thus, creativity in dance is a social phenomenon (Łucznik, 2015). As Csikszentmihalyi (1999) notes, “*The underlying assumption is that an objective quality called ‘creativity’ is revealed in the products and that judges and raters can recognize it*” (p. 314). Csikszentmihalyi (2014) argues that the interaction between three elements of a system constitute creativity. A culture contains symbolic rules for creativity, the individual brings that creativity into the domain, but creativity is only brought to fruition when experts from that domain recognize the creativity. Recognition of creativity occurs in contemporary dance education (for example, the ability to demonstrate creative engagement in improvisation is a typical entry requirement to higher education dance training), subsequent student assessments, and in reviewing professional work. Although experts are imperative to real-world creativity assessment, non-experts also play a role in the day-to-day sustenance of creative careers, and varying levels of expertise or knowledge may predict differences in assessment of creativity (Hong and Lee, 2015).

Since participation in contemporary dance is an increasingly popular recreational, educational and professional pursuit, one could argue that the audiences who engage with and see this creativity should also be increasing too. Burrows (2010) highlights that contemporary dance audiences seek novelty, but alternative research has also shown that some less experienced contemporary dance audiences report confusion, failure to understand the choreographic intention, and lack of enjoyment (Stevens et al., 2007, 2009; Van Dyke, 2010). Audiences of varying levels of expertise, levels or types of training, may, therefore, assess creativity differently. Research in dance indicates that non-expert dance audiences may fail to understand the meaning behind contemporary dance, perhaps because contemporary dance is detached from the ‘magic’ seen in dance which makes use of popular music, costumes and staging (Stevens et al., 2009). Contemporary dance has not become rooted in modern westernized culture in the same way other art forms or classical ballet have. For example, a dance director reports that his audiences mainly consist of friends, family or supporters of those directly involved in the performance rather than members of the public (Van Dyke, 2010). Contemporary dancers are often dressed in plain, everyday clothes or speaking directly to audiences; the movement is often pedestrian and effortless, or, hugely effortful. Often, dancers create movement without music, and the music is added later in the choreographic process. Thus contemporary dance may be a particularly unique and ripe area for novel research into creativity, and given this previous research we were interested in the broad role of expertise and understanding of contemporary dance in assessing creativity.

Williams et al. (2016) note that despite the growth of the psychology of creativity over the last 25 years, in particular, many fundamental complexities remain. One such challenge is finding appropriate methodologies for investigating previously underresearched domains of creativity. Problem solving approaches are perhaps the most common methodology seen in psychology research, where ‘creativity’ lies in the process or means by which an individual arrives at a solution (Lubart, 2001). Problem-solving measures predominantly investigate insight, also known as the ‘aha moment’ (e.g., the Remote Associates Tests, Wallas, 1926; Mednick and Mednick, 1971; Runco and Jaeger, 2012). In these tests, problem solving involves a two-stage process of divergent and convergent thinking; restructuring the problem by reframing one’s mental approach, to find the one appropriate answer (Guilford, 1956). A small number of research studies have used problem-solving approaches to dancers’ creativity, using measures of divergent thinking (the ability to produce multiple responses to a problem) which is considered the ‘backbone of creativity assessment’ (Runco, 2014, p. 14). Stinson (1993) found students in Chinese dance education were significantly less creative (in divergent thinking) than a non-dancing control group. Fink and Woschnjak (2011) found differences in divergent thinking across contemporary, ballet and jazz dance, suggesting that creativity differs within dance genres. These studies suggest that differences in creativity occur at the microdomain level of dance, yet their generalized approach to assessing creativity may limit their usefulness.

There are reasons why traditional divergent thinking measures may be of limited use for choreographic creativity. Most importantly, some criticize the problem-solving approach to creativity assessment for constituting just one type of creativity, which assumes domain generality of the cognitive processes (i.e., attention, perception, memory, language, and intelligence) underpinning creativity (Kaufman and Baer, 2004; Runco, 2014). At this level, creativity is a nomothetic process shared by, and accessible to, all humans (Simonton, 1999; Glăveanu, 2010). This generalist perspective arguably lacks sensitivity to the individual nuances of creative specialization that manifest in different ways across different fields (Baer, 1998; Feist, 1998; Hu and Adey, 2002; Julmi and Scherm, 2015). Divergent thinking tests may assess only narrow ranges of ability and may not be conclusive about measuring ‘creativity’ itself. Instead, they indicate abilities related to creativity, which may not be as relevant in specialized domains (Amabile, 1982; Baer and McKool, 2009). Thus it is important also to develop methodologies that are sensitive to the individual nuances of creativity in each domain.

Choreographic creativity, for example, implicates embodied cognition: cognitive processes are rooted in physical interaction with the world (Wilson, 2002; Stevens and McKechnie, 2005). Embodiment emphasizes both physical exploration and knowledge (Kogan, 2002). Dancers understand the intention and action of others moving in the same space and use the body for problem-solving, demonstrating creativity by thinking with the body (Kirsh, 2011). Choreographic creativity uses both awareness of kinesthetic knowledge and experience in/through the body and explicit knowledge of the external world; cognition is situated (Risner, 2000; Kirsh, 2010, 2011). Thus creativity in dance is a process of using the body in novel ways in response to a task and the ability to successfully and fluidly link body positions into a developed sequence (Stevens et al., 2001; Stevens and McKechnie, 2005; Kirsh, 2011). These processes use memory, language and perception as well as space, time, motion and physical expression, with decreased emphasis on verbal and greater emphasis on nonverbal communication (Bläsing et al., 2010; Thomson and Jaque, 2017). Hagood (2001) writes that dance, in general, is “an extremely complex experience to attempt to measure” (p. 27). However, embodiment and process are critical, which differs starkly from the pen and paper medium emphasized in time-limited psychology measurement traditions; thus studying creativity in dance would be wise to use dance in its natural movement based form.

One of the most widely advocated domain specific means of assessing situated creativity is the Consensual Assessment Technique (CAT; Amabile, 1982). The CAT is popular in psychology since it is unrelated to any specific creativity theory, meaning that its use is broad and relevant to any domain of creativity (Baer and McKool, 2009). In the CAT methodology, experts assess creativity using an implicit understanding within their specific domain (Amabile, 1982; Amabile and Pillemer, 2012). Similarly, assessors in dance use an implicit creativity definition to assess. For example, it is common to obtain mean scores from panels assessments during improvisation at an audition.

However, the CAT has some challenges. Namely, there are no clear guidelines for implementation, and many variations have been used to investigate specific domains. It is a process of obtaining evaluations from raters without using a formal tool or needing to provide explicit criteria against which creativity must be assessed. Conventionally, it is expected that raters should share some common understanding of the domain to support a consensus.

Although a large body of research has investigated audience responses to classical dance as a performance (See Calvo-Merino et al., 2005; Reason and Reynolds, 2010), there is a paucity of research into contemporary dance audiences which focuses on perceptions of creativity. Research has been undertaken to explore the associative and affective results of performance (e.g., Stevens and McKechnie, 2005), but no research has considered audience evaluations of creativity using the psychology of creativity methods such as the CAT. Research using the CAT supports that expert and non-expert creativity assessments of poems differed significantly different, with expert raters giving a higher rating than non-experts, thus is a suitable methodology for investigating choreographic creativity (Kaufman et al., 2008). Kokotsaki and Newton (2015) suggest a continuum of insider-outsider status that potential creativity assessors have, depending on their expertise and experience. Therefore, using a simple dichotomy of expert or non-expert may be too restrictive, particularly in dance where individuals gain experience through doing, making and watching.

The role of creativity has been the subject of considerable interest in psychology research but is yet to be explored in depth in dance within a scientific framework. Therefore, the purpose of this research was to establish an understanding of expertise on the attribution of creativity in contemporary dance choreography. We aimed to recruit a large sample of assessors to judge choreographic creativity of contemporary dance. Informed by the method of the CAT, we used a quantitative methodology to assess the impact of expertise in assessing creativity in contemporary dance to rate video clips of student choreographies (Amabile, 1996). Additionally, we collected measures of perceptions of technical ability, liking and ability to find meaning, as previous research has indicated that non-experts use these variables to assess creativity (e.g., Kozbelt, 2004; Glass and Stevens, 2005).

## Materials and Methods

### Participants

#### Choreographers

Students (*n* = 24; male *n* = 6, female *n* = 18, mean age = 20.2 years; *SD* = 1.6 years) studying in the 1st year of a BA Contemporary Dance at Trinity Laban, a leading UK Dance Conservatoire, consented to participate in the research. Students entered onto the degree having been assessed for both technical and creative skill at audition (evaluated through a panel marked improvisation), thus had been selected onto the program for their creative potential. Their dance training consists of technique classes in Contemporary Dance (such as Graham and Cunningham) and Ballet, as well as Choreography classes focused on developing processes of exploratory non-stylistic ways of moving from within the body. Students take additional modules in performance and contextual studies. Students were all members of the same choreography class, taught by the same teacher, and had been randomly allocated to this teacher’s class at the start of the academic year (from four possibilities).

#### Creativity Raters

We recruited creativity raters (*n* = 1084) from a variety of levels of expertise to the research. After data screening and cleaning, the final sample size was 850 raters (female *n* = 682, male *n* = 158, other *n* = 10). Participants ranged in age from 18 to 77 years (*M* = 31.6, *SD* = 12.9). We created dummy variables using the nine categories of experience and expertise seen in **Table 1**, whereby an individual who’s answer was ‘No’ is coded as the reference category of ‘0’, and an individual who’s answer was ‘Yes’ to any degree of experience is coded as ‘1’. The employment categories were answered qualitatively and coded by the first author as ‘No’ or ‘Yes’. An overview of rater experience and expertise in dance and creativity are shown in **Table 1**.

**Table 1:** Participant experience and expertise in dance and creativity.

Experience/expertise	No (N)	Yes (N)
Experience in child/adult dance classes	*n* = 166	*n* = 684
Experience in child/adult contemporary dance classes	*n* = 444	*n* = 406
Current/previous attendance at the dance institution	*n* = 763	*n* = 87
Experience in watching live contemporary dance	*n* = 504	*n* = 346
Experience in choreographing dance	*n* = 262	*n* = 588
Experience choreographing contemporary dance	*n* = 605	*n* = 245
Employed in any creative domain	*n* = 383	*n* = 467
Employed in an artistic, creative domain	*n* = 483	*n* = 367
Are you an expert in creativity?	*n* = 714	*n* = 136

### Measures

#### Video Stimuli

We obtained videos of a short solo choreography (*n* = 23; duration 172–194 s), which were created for the students’ choreography module assessment. The choreography was danced by a classmate of the student, rather than the choreographer themselves. We filmed the choreographies in a mirrorless dance studio in natural lighting to standardize the videos and remove confounding variables relating to production. We used a wide shot of the dance studio which replicated a head-on audience view. All dancers dressed in plain, dark colored practice clothes. An audio-visual expert removed the music and added a fade in and out at the start and end of each piece.

#### Creativity Ratings

Creativity was assessed using a seven-point Likert scale (*How creative did you think the piece was?*; 1. Not at all creative – 7. Very creative) informed by the method of the CAT (Amabile, 1983). In addition to the target question, participants answered three additional questions; *How much did you like the piece?* (1. Not at all – 7. Very Much); *How technically skilled did you think the dancer was?* (1. Not at all technically skilled – 7. Very technically skilled); *How able were you to find meaning in the piece?* (1. Not at all able to find meaning – 7. Very able to find meaning).

### Procedure

We obtained institutional ethical approval. Following this, a choreography teacher provided initial consent to approach her first-year choreography students to provide choreographic material for creativity assessment in the research. The contemporary dance students consented at the end of a timetabled choreography class, 2 weeks before their choreography assessment. Each student’s assessed work was a three-minute solo performed by a peer in the same class, so each student consented once for the inclusion of their choreography and a second time as a performer in a peer’s work.

On the day of the assessment and filming for the research, each participant provided secondary verbal consent to confirm his or her inclusion. One participant was injured so did not undertake her performance, resulting in 23 videos. We embedded the clips into an online survey via a video hosting site. Snowball sampling was used to recruit creativity online raters through online platforms, social media and email groups. A variety of groupings were targeted, including those with experience in dance, those with experience in creative fields, and those who had no experience in dance and/or creativity. Participants completed comprehensive demographic questions to provide information about their background and training in dance, creativity and the arts. They then watched a randomly selected video, before completing the four assessment scales (creativity, liking, technique and meaning), which appeared in a random order. Each participant had the option to watch as many clips as they wished to, before completing the four scales at the end of each piece.

After 6 weeks, we had obtained sufficient data. Data were downloaded to Microsoft Excel and cleaned and screened, where participants with missing data or insufficient information were removed. We then transferred data into the Statistical Package for Social Sciences Version 23 (IBM Corp, 2016), and undertook preliminary analyses of variance and correlation. We conducted main analyses using the LAVAAN package (Rosseel, 2012) within R version 3.2 (R Core Team, 2015). A repeated measures linear mixed model was used to predict creativity score and determine the impact of experience and expertise at the nine levels. We used a repeated measures mixed model as it is suitable for missing data, therefore allowing for the variation in the number of videos observed.

## Results

### Descriptive Statistics

The numbers of videos viewed by each of the 850 creativity raters ranged from one to 21 videos (*M* = 2.53, *SD* = 2.63). In total, we obtained 2153 individual ratings with between 81 and 102 creativity ratings on each video (*M* = 91.61, *SD* = 6.37). Descriptive statistics of overall ratings from the 23 videos are shown in **Table 2**.

**Table 2:** Descriptive statistics of creativity, likeability, meaning and technique ratings.

	Min.	Max.	Mean	*SD*	Skewness	Kurtosis
Creativity	1.0	7.0	4.62	1.44	−0.45	−0.17
Likeability	1.0	7.0	4.13	1.61	−0.21	−0.67
Meaning	1.0	7.0	3.86	1.64	−0.17	−0.78
Technique	1.0	7.0	4.97	1.36	−0.55	−0.05

### Preliminary Analyses

We undertook a series of one-way ANOVAS to determine a difference in the mean ratings of the videos. Creativity [*F*(22,2130) = 6.85, *p* < 0.001], likeability [*F*(22,2130) = 5.90, *p* < 0.001], meaning [*F*(22,2130) = 4.77, *p* = < 0.001] and technique [*F*(22,2130) = 11.44, *p* < 0.001] all showed significant variation in scores between videos.

Next, Pearson’s correlation analyses were conducted to obtain an understanding of the relationships between creativity, likeability, technique and meaning. **Table 3** shows significant moderate to strong positive correlations between all four variables, suggesting that people rate contemporary dance highly on creativity when it is also perceived as liked, well understood and well executed.

**Table 3:** Pearson’s correlation coefficients for creativity, likeability, meaning and technique ratings.

	Creativity	Likeability	Meaning
Creativity			
Likeability	0.71^∗^		
Meaning	0.60^∗^	0.67^∗^	
Technique	0.62^∗^	0.57^∗^	0.45^∗^

*^∗^Correlation is significant at the 0.05 level (2-tailed).*

### Repeated Measures Linear Mixed Model

A colleague of the authors’ who was blind to the purpose of the research coded a random sample of 50 participants’ qualitative employment responses ‘Employed in any creative domain’ and ‘Employed in an artistic creative domain’ to assess the reliability of the expertise and experience coding seen in **Table 1**. A positive inter-rater reliability (IRR) correlation = 0.83 was achieved. According to Cohen’s Kappa statistic, an IRR of greater than 0.8 indicates a very good level of reliability between raters (McHugh, 2012).

We entered each of the experience or expertise categories in to the repeated measures linear mixed model as a fixed effect. Contemporary choreographic experience significantly predicted creativity, *F*(1,2052.33) = 6.61, *p* < 0.001, as did self-attributed creative expertise *F*(1,2032.13) = 17.82, *p* < 0.001, but none of other categories were significant. In those with contemporary choreographic experience, creativity was rated higher compared to the reference group [*b* = 0.24, *t*(2067.48) = 2.71, *p* < 0.05 (95% CI = −0.044 to 0.39)]. In those with self-attributed creative expertise, creativity was rated lower compared to the reference group [*b* = −0.13, *t*(2032.13) = −4.44, *p* < 0.001 (95% CI = −0.52 to −0.19)]. Next, video was entered as a random effect. Both the intercept (*b* = −0.19, *Wald Z* = 32.56, *p* < 0.001) and video were significant (*b* = −0.12, *Wald Z* = 2.81, *p* < 0.05), indicating that slopes were significantly different across the 23 videos.

## Discussion

We explored the role of experience and expertise in assessing choreographic creativity, using a novel online methodology that facilitated dance specific research. 850 assessors assessed creativity in 23 individual contemporary dance choreographies. Assessor experience and expertise were sampled from a continuum of expertise from those who had never taken a dance class to professional choreographers. The results demonstrate the impact of both dance specific experience and broader creative expertise in the assessment of choreographic creativity.

The results show that when an individual has experience in choreography, they rate creativity higher. That is, one needs experience in the choreographic process to judge a piece to be more creative. This supports the idea by Corazza (2016) that creativity is related to an ability to see the potential expression of a process. This is in line with the emphasis on the creative process in dance pedagogy (Butterworth, 2004; Farrer, 2014), yet suggests that this emphasis may be preventing those who do not have experience of choreography from identifying creativity. Our findings suggest that this level of expertise is essential in evaluator selection; experience in physically dancing or watching contemporary dance does not lead an individual to rate creativity higher. Instead, experience in knowing the process of making dance allows an individual to judge a piece as more creative.

These findings have implications with regards to accessibility of contemporary dance, in suggesting that training in dance *per se* does not necessarily facilitate an understanding of choreographic creativity, but that only those who learnt to make dance understand and rate higher. The level of expertise suggested by our findings regarding creativity is more specific than that which has been reported in the literature on dance performance, even beyond those studies involving fMRI recordings of audience responses (e.g., Calvo-Merino et al., 2005). Here, physical participation has led to significant differences in brain activity when watching dance. However, our findings indicate that experience of making or choreographing, beyond physical participation in dancing, impacts creativity assessment (e.g., Calvo-Merino et al., 2005).

The results of the correlational analyses showed that creativity score is related to choreography that the evaluator likes, can find meaning in, and is performed by a dancer whom the evaluator perceives as technically skilled. Collectively, these correlational results indicate that an audience evaluates creativity in line with subjective elements which go beyond the criteria which underpin problem-solving tests such as the RAT (Mednick and Mednick, 1971) and TTCT (Torrance, 1974). Standard creativity tests previously used in dance, operationalise creativity by the ability to rapidly produce a large number of infrequent responses (e.g., Fink and Woschnjak, 2011). Two critical elements of creativity underpin most theoretical and research-based definitions) *originality* or *novelty* and b) *usefulness* or *appropriateness* (Stein, 1953; Barron, 1955; Amabile, 1983; ). This dualistic criterion remains the most commonly accepted definition of creativity (Runco and Jaeger, 2012). Since creativity correlated highly with making meaning of the piece, one could argue that those who rated higher in the contemporary dance choreography subgroup had a clearer insight into the meaning of the work, because they had experience of the process and understood intention. Creativity in the arts may be assessed concerning intention at the moment of creation, with a proposition that it is intentionality rather than novelty which is vital (Kharkhurin, 2014; Weisberg, 2015). In turn, this supports previous authors who have discussed the lack of outsider dance audiences and the failure to understand contemporary dance (Van Dyke, 2010).

A second finding was that scores by those who self-assigned themselves as creative experts were lower than those who did not. This supports the value of the chosen method, and that asking judges to self-select whether they are an expert may be valuable when seeking to recruit judges. Experts will have had considerably greater exposure to creativity and therefore do not consider the work to be as creative; there is some interaction of expertise at this level, yet cause and effect cannot be established.

The implications for these findings are numerous when discussing the need for widening audience engagement in contemporary dance. These findings may imply a need for educating audiences about creative processes underpinning the dance product. Glass and Stevens (2005) note that ‘*Priming audience members about a particular work should assist them to engage with the work at a greater level of understanding*’ (p.17). Educating an audience about the creative process might bridge the gap between the audience’s understanding of creativity in dance and subsequent enjoyment of the work. This may be particularly true in an art form where the emphasis is on the process and the dancer’s experience of making or creating a dance for the dancer’s enjoyment (Lavender, 2009).

Importantly, the results of the analyses showed variation in the mean ratings of the videos, demonstrating that the snowball sampling method does not neutralize differences; that is, a varied audience collectively distinguish varying levels of creativity. Using a simple Likert scale for the CAT is therefore advocated as a simple yet effective measure of creativity. We recognize that there are numerous ways of implementing the CAT and the present research was a considerable variation on the original. The use of this variation was beneficial since it is arguably the *only* available research methodology for creativity which is not inherently tied to a theory of creativity but facilitated a means of assessing dance specific creativity (Baer and McKool, 2009). The methodology assessed the manifestation of creativity through the body (Kirsh, 2010 and without pen and paper tests, while focusing on product also increased validity.

It is of note to consider the relationship between the choreographer and the dancer who is performing the work. Whilst our intention was to assess the choreographer’s creativity, one could argue that the audience perception may also be related to the performer’s creative interpretation of the movement, in the same way that it is related to their technical skill. Thus an additional facet in dance may be the dancer’s ability to communicate and interpret the choreographic interpretation which is as important as the choreographer’s creative skill at constructing the work (Smith-Autard, 2014).

The study is strengthened by the inclusion of 23 videos and a large sample of respondents, allowing a more substantial variation of scores to be given and to facilitate a broad audience, which is more reminiscent of real-life choreographic settings. Future research should endeavor to establish reliability amongst experts in dance specific creativity which is solely reliant on expert opinions, such as auditions. The present research was not intended to undertake IRR correlation analysis; however, IRR between experts has been highlighted as methodologically important (Kaufman et al., 2008; Haller et al., 2011). Furthermore, there is debate regarding the width of the Likert scale, with no consistent recommendations, aside from to include a neutral point. Thus, findings are not comparable across studies. However, in sum, although the method underpinning the CAT may be perceived to lack methodological stability, the breadth of application and validity has been demonstrated.

We had 87 (of 850) participants who currently/previously attended the institution, so we added ‘current/previous attendance at the institution’ as a predictor. This was not significant, thus did not impact on creativity ratings. Therefore the possibility of this as a confound was deemed to be minor, since only a small number of participants were potential classmates and this did not have a significant impact. In addition, although we did not ask whether the viewer knew the performer in the video, the video appeared randomly, so if they knew any performer, there was a 1 in 23 chance of them knowing the performer on video 1, 1 in 22 chance for video 2 and so forth. Since the average views were 2.5, the chances of knowing the performer were again relatively small.

The online methodology and use of snowballing enabled meaningful participant diversity, which was also sensitive to differences both in expertise and in evaluations of the videos. We recognize that snowballing can result in the loss of crucial information over participants, however, for the present research it facilitated a meaningful audience-like participant set. The use of such an online evaluation might facilitate repeated testing over time. Previous efforts to research dancers’ creativity focused on domain-general measures and tended to be cross-sectional in nature; longitudinal research looking at the impact of the environment or training on dancers’ creativity has not yet been possible (e.g., Kalliopuska, 1989; Stinson, 1993; Fink and Woschnjak, 2011). Although we note that there are limitations of online methodologies, such as being unable to establish reliability between evaluators (as is common in the original version), the results of the study support the viability of an online snowball sampling method to recruit both experts and non-experts. In particular, the effectiveness of adapting the CAT for research purpose is advocated.

The present online adaption has strength in its flexibility for use across many unique domains of creativity. Thus, by assuming neither domain generality nor specificity, it is a method which could be replicated using any creative performances or artifact across many arts such as music, or visual art, allow recruitment of both large samples of creative works and raters. In this variation, a methodological strength was that unknown to the raters, the individual performing the work was not the creator. Future research within the domain of dance should continue to use the CAT in its most original form, aiming to establish reliability between assessors in real life creative performance scenarios such as an audition, to understand selection methods, as well as evaluation of students in choreography and improvisation courses.

## Conclusion

This research aimed to understand the role of expertise in assessing creativity in choreographic creativity. A secondary aim was to use a large scale online methodology which went beyond the pen and paper problem-solving approaches which have predominated the literature. The use of choreographic videos allowed the expression of embodied creativity and recruitment of a large audience with varying degrees of expertise and experience in dance. The results showed that personal experience of the creative process increased ratings of creativity, while creative experts rated creativity lower. The use of online methodologies for assessing creativity is advocated across multiple domains of creativity.

## Ethics Statement

This study was carried out in accordance with the recommendations of Trinity Laban Ethics Guidelines and the BPS Code of Ethics. The protocol was approved by the Trinity Laban Conservatoire of Music and Dance Ethics Committee. All subjects gave written informed consent in accordance with the Declaration of Helsinki.

## Author Contributions

LC led the work carried out on the paper, including the writing, research design, data collection, and analysis. JM supported the development and undertaking of statistical analyses. NLS assisted in developing the methodology. ER and JM were Ph.D. supervisors of the work.

## Conflict of Interest Statement

The authors declare that the research was conducted in the absence of any commercial or financial relationships that could be construed as a potential conflict of interest.
